# Clinical characteristics of tracheobronchial *Talaromyces marneffei* infection in non-HIV-infected patients in South China

**DOI:** 10.1080/07853890.2023.2276310

**Published:** 2023-11-15

**Authors:** Mianluan Pan, Gaoneng Fang, Fei Zheng, Fanhai Lin, Wen Zeng, Ye Qiu, Jiehua Deng, Xiangmei Chen, Jianquan Zhang

**Affiliations:** a Department of Respiratory and Critical Care Medicine, The Eighth Affiliated Hospital of Sun Yat-Sen University, Shenzhen, Guangdong, China; b Department of Respiratory and Critical Care Medicine, The First Affiliated Hospital of Guangxi Medical University, Nanning, Guangxi, China; c Department of Respiratory Medicine, Minzu Hospital of Guangxi Zhuang Autonomous Region, Guangxi Medical University, Nanning, China; d Department of Respiratory and Critical Care Medicine, The First People’s Hospital of Huaihua City, Huaihua, Hunan, China; e State Key Laboratory of Respiratory Disease, National Clinical Research Center for Respiratory Disease, Guangzhou Institute of Respiratory Health, The First Affiliated Hospital of Guangzhou Medical University, National Center for Respiratory Medicine, Guangzhou, China

**Keywords:** *Talaromyces marneffei*, tracheobronchial, bronchoscopy, amphotericin B

## Abstract

**Objectives:**

Tracheobronchial *Talaromyces marneffei* (*T. marneffei*) infections among non-HIV-infected patients are rare. To improve understanding, we analysed the clinical features, immune mechanisms, treatment, and prognosis.

**Methods:**

Data on hospitalized patients with tracheobronchial *T. marneffei* infections from September 2013 to May 2022 were collected. The clinical and imaging features were analysed.

**Results:**

Nineteen patients were enrolled, with a median age of 52 years (45–62 years). The most common symptoms were cough, expectoration, fever, weight loss, and anaemia. The total white blood cell and neutrophil counts, erythrocyte sedimentation rate, C-reactive protein, procalcitonin and globulin were increased, and the serum albumin levels were decreased. Chest CT manifestations included patchy shadows, masses, obstructive atelectasis, cavities, pleural effusion, and hilar and mediastinal lymphadenopathy. The fibreoptic bronchoscopy findings included masses, polyps or nodules with mucosal oedema, hypertrophic bulges, lumen stenosis or obstruction, and purulent secretions. *T. marneffei* infection was confirmed in 10 patients by positive culture, in five by both culture and metagenomic next-generation sequencing (mNGS), in two by mNGS, in one by culture and pathology and in 1 by histopathology. BALF (15/19, 78.9%) had the highest culture positive rate, followed by sputum (3/19), bronchial mucosa (1/1), lung biopsy (1/2); 36.8% of the patients were coinfected with other pathogens. For induction therapy, 7, 6, 2, and 4 patients received voriconazole, amphotericin B, voriconazole combined with amphotericin B, and fluconazole therapy, respectively, and 26.3% received treatment combined with nebulization and/or administration of amphotericin B under fibreoptic bronchoscopy. Four patients were treated for underlying diseases or coinfection, 31.6% were cured, 42.1% improved, and 26.3% died.

**Conclusions:**

*T. marneffei* infection is common in the tracheobronchial airway tissue or secretions, and bronchoscopy has important diagnostic and treatment value. Antifungal therapy, including systemic therapy, involves triazoles and amphotericin administration, and aerosol inhalation and administration of amphotericin B under bronchoscopy are important.

## KEY MESSAGES

- *T. marneffei* infection involving the tracheobronchial region in airway tissue or secretions is high, and bronchoscopy has important value in diagnosing and treating these patients
- The use of triazoles and amphotericin and the aerosol inhalation and instillation of amphotericin B under bronchoscopy are essential to antifungal therapy.

## Introduction

Talaromycosis marneffei is an invasive fungal disease caused by *Talaromyces marneffei* (*T. marneffei*) infection. In Southeast Asia, *T. marneffei* is the third most common presenting illness in AIDS patients. Talaromycosis marneffei is categorized as either localized or disseminated. *T. marneffei* disseminates *via* the blood or the lymphatic system throughout the body, affecting the lung, liver, spleen, lymph nodes, skin, and other tissues and organs. The respiratory system is the earliest and most commonly involved system, and respiratory involvement accounts for 66.7% of cases [1]. It is suggested that the respiratory route may be one of the transmission routes of *T. marneffei* infection in humans. However, clinical reports of tracheobronchial *T. marneffei* infections are very rare. Moreover, *T. Marneffei* infection with respiratory system lesions is often misdiagnosed as pulmonary tuberculosis and lung cancer. Long-term anti-tuberculosis treatment leads to refractory pneumonia and systemic spread. The clinical manifestations we observed in a patient whose case we reported for the first time were very similar to those observed in patients with bronchial tuberculosis, bronchial tumours, and other fungal infections. Due to delayed diagnosis and treatment, extensive lysis and destruction of the airway result in fatal asphyxia [2]. Therefore, it is very important for clinicians to be familiar with tracheobronchial Talaromycosis marneffei to improve the rate of early recognition, facilitate the initiation of treatment, and improve patients’ overall quality of life.

## Materials and methods

### Study population

We retrospectively analysed patients with tracheobronchial *T. marneffei* infections between September 2013 and May 2022 at The First Affiliated Hospital of Guangxi Medical University. The medical records included demographic information (sex and age), clinical characteristics, laboratory findings, and clinical outcomes, and data on these variables were collected.

**Figure 1.**
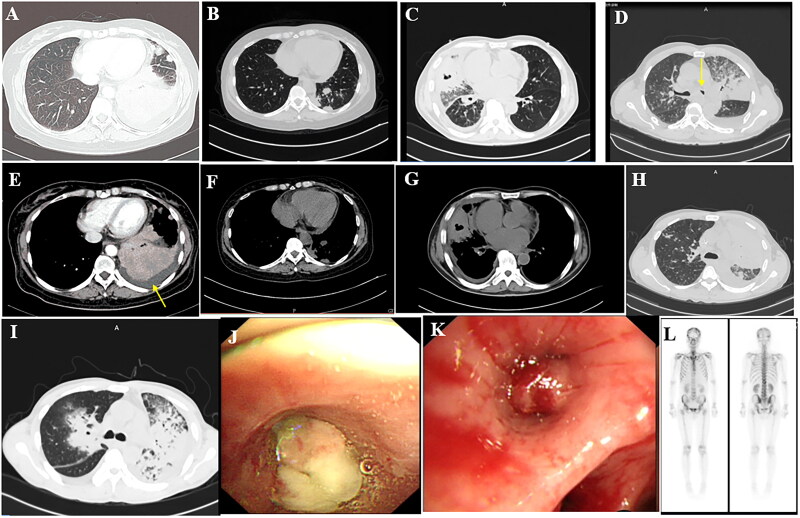
(A,E) Shadow of the left lower lobe, left pleural effusion; (B,F) Nar shadow of the left lower lobe (C,G). The middle lobe of the right lung is incomplete, with internal bronchial inflation and cavity, and right pleural effusion. (D) Irregular mass shadow of the left superior lobe and narrow left main bronchus. (H) Diffuse density increase shadow in the left lung and inflatable bronchial shadow. (I) large solid lesions, even on the left, with bronchial inflation, (J) The left main bronchial terminal mass completely blocked the official lumen, indicating attachment of necromass. (K) The opening of the right middle lobe is completely blocked. (L) The skull is concentrated in multiple parts, including the 12th thoracic vertebra, the first lumbar spine, multiple heel ribs, and a small tablet imaging agent with concentrated poly shadow.

According to the strict regulations on a retrospective study of the Ethics Committee of the first affiliated Hospital of Guangxi Medical University, written informed consent was obtained from all patients (signed by the patient or their immediate family) prior to the study, and the ethic approval number was 2022-E343-01 (Figure 1).

### Inclusion and exclusion criteria

The inclusion criteria were as follows: (i) HIV-negative status and (ii) Talaromycosis marneffei patients with airway lesions under bronchoscopy and respiratory specimens from which *T. marneffei* was isolated.

The exclusion criteria were as follows: (i) Talaromycosis marneffei without respiratory involvement, (ii) Talaromycosis marneffei with clinical manifestations of respiratory but no evidence of positivity for *T. marneffei* infection in respiratory specimens, or (iii) patients with lung tissue involvement but no tracheobronchial involvement by bronchoscopy.

### Methods used to diagnose *T. marneffei* infection

To diagnose *T. marneffei* infection, microbiological or pathological findings identified from clinical specimens consistent with any of the following manifestations were utilized: (a) isolation of pathogens from culture; (b) visible detection of fungi (intracellular yeast-like or sausage-shaped cells measuring 2–3 mm in diameter with transverse septa.) by microscopy after Periodic Acid-Schiff or Wright staining. Finally, as a molecular biological method for confirming the diagnosis, metagenomic next-generation sequencing (mNGS) was applied for the early diagnosis of *T. marneffei* infection. When *T. marneffei* infection was further confirmed by the traditional methods mentioned above, the diagnosis was confirmed. When only the mNGS analysis of tissue samples confirmed the presence of *T. marneffei*, other pathogens were excluded and antifungal treatment was effective, these patients were also considered to have a confirmed diagnosis of Talaromycosis marneffei infection in the analysis.

### Anti-IFN-γ autoantibody (AIGA) assay

Serum samples were obtained under sterile conditions before the patient received antimicrobial therapy and during the active stage of the infection. Serum was separated by centrifugation at 3000 rpm for 10 min, and the samples were diluted 16-fold. The detection of AIGA in the serum was performed using an enzyme-linked immunosorbent assay kit (Cloud-Clone Corp., Wuhan, China) according to the manufacturer’s protocols. The concentration of anti–IFN-γ in the sample was then determined by comparing the optical density of the sample to the standard curve [3].

## Results

### Demographic data and clinical characteristics

During the 9-year study period, 210 patients were diagnosed with *T. marneffei* infections, and 126 patients had respiratory system infections. In addition, bronchoscopy was performed in 59 patients; finally, 19 patients with tracheobronchial *T. marneffei* infections were selected. All 19 patients were born in Guangxi Province and stayed in *T. marneffei* endemic areas in southern China. The study population included 11 men and 8 women with a median age of 52 years (range 45–62 years). Their occupations included being a farmer (*n* = 14), retired (*n* = 3), self-employed (*n* = 1), and unemployed (*n* = 1). Comorbidities included AIGA-associated immunodeficiency (*n* = 11), type 2 diabetes mellitus (*n* = 2), pulmonary tuberculosis (*n* = 2), breast cancer, Sweet’s syndrome, erythema nodosum and ankylosing spondylitis (*n* = 2). Seven patients were misdiagnosed with breast cancer, 4 with pulmonary tuberculosis, and 2 with bacterial pneumonia. The time from the onset of clinical symptoms to the diagnosis of *T. marneffei* infection was 55–224 d (median: 133 d). Common clinical features of *T. marneffei* infection included cough, expectoration, fever, weight loss, and anaemia, followed by chest pain, fatigue, shortness of breath, rash, anorexia, and bone pain/arthralgia haemoptysis (Table 1).

**Table 1.** Characteristics of the 19 patients with tracheobronchial *T. marneffei* infection.

ID	Comorbidity	Misdiagnosed diseases	Days require for diagnosis	Diagnostic methods	Clinical manifestations	Chest CT	Fibreoptic bronchoscopy findings	ECT	First-line therapy	Sequential therapy	Outcome
P1	TB	None	196	Lung, BALF, sputum(Fic)	Fever, night sweats, cough, expectoration, chest pain, shortness of breath, fatigue, anaemia, muscle and joint pain, weight loss, Swollen lymph nodes	Patchy opacities, obstructive pneumonia, pleural effusion	Hypertrophy of left bronchial mucosa, stenosis, blockage of right middle bronchus, purulent secretions	Bone defect of right femur and humerus	FLC 0.4 g/d for 2 weeks	ITC for 1 year	Cured
P2	None	Bacterial pneumonia	262	BALF, Purulent discharge(c)	Fever, cough, expectoration, fatigue, rash, Swollen lymph nodes	Patchy shadows, hilar and mediastinal lymphadenopathy, right pleural effusion	The right middle lobe opening was blocked by a tumour, the right lower lobe basal opening was a tumour, and the surface of the bronchial mucosa was uneven	Skull, clavicle, right femur radionuclide concentration, right femur bone destruction	FLC 400 mg/day for 18 days then change to VCZ 200 mg every 12 h improved after 2 weeks	VCZ for 6 months	Improved
P3	Lung cancer	None	61	Bronchial mucosa(h)	Fever, cough, expectoration, weight loss, swollen lymph nodes	Consolidation shadow, left pleural thickening, hilar mediastinal lymphadenopathy, left pleural effusion	Nodular bulging and narrowing of the left lower lobe bronchial opening	ND	VCZ 200 mg every 12 h for 2 w	ITC for 9 months	Cured
P4	Type 2 diabetes mellitus	TB	234	BALF(c)	Cough, expectoration, fatigue, weight loss, swollen lymph nodes	Patches, cavities, streak shadows, obstructive pneumonia, right pleural thickening, right pleural effusion	Right middle lobe mucosal oedema, open nodular mass	ND	AMB 0.6 mg/kg/day for 2w	ITC for 4 years	Improved
P5	None	TB	133	BALF(c)	Fever, cough, expectoration, shortness of breath, dyspnoea, fatigue, anorexia, weight loss, rash, pleural effusion	Patchy shadows, consolidation shadow, nodular shadow, bilateral pleural effusion	One nodule anterior and superior to the carina, mucosal congestion and oedema, two nodules on the posterior wall of the right main bronchus, mucosal oedema of the left upper bronchus, stenosis of the left lower bronchial opening	ND	VCZ 200 mg every 12 h for 2 w	VCZ for 6 months, ITC for 7 months	Cured
P6	None	TB	301	Tracheal tumour tissue, BALF(c)	Fever, cough, expectoration, chest pain, shortness of breath, anorexia, arthralgia, bone pain, swollen lymph nodes	Patchy shadows, consolidation shadow, nodular shadow, obstructive pneumonia, hilar and mediastinal lymphadenopathy, left pleural effusion	The opening of the left lower lobe is narrow and polypoid protrusions can be seen on the interlobar ridges	Left 7th rib fracture	AMB 0.6 mg/kg/day for 1 m	ITC for 7 months	Cured
P7	None	None	44	BALF(c)	Fever, chills, cough, weight loss, swollen lymph nodes	Patchy shadows, streak shadows, bilateral pleural thickening, hilar and mediastinal lymphadenopathy, bilateral pleural effusion	Purulent discharge from left main bronchus, swelling of right middle bronchus, mucosal hyperaemia and oedema	ND	FLC 400 mg/day for 10days, then change to AMB 0.6 mg/kg/day for 2w	ITC for 1year	Death
P8	TB	Bacterial pneumonia	30	BALF(c), skin lesion(c + h)	Cough, expectoration, chest tightness, fever, dyspnoea, fatigue, skin mass, pleural effusion	Patchy, cord-like high-density shadows, pleural thickening, mediastinal lymphadenopathy, pleural effusion, pericardial effusion	The proper branch of the left upper lobe and the opening of the left lower lobe is narrowed, mucosal congestion, oedema, and a small amount of serous secretions	ND	VCZ 200 mg every 12 h for 2 w	ITC for 3 years	Improved
P9	An anti-IFN-γ autoantibody-associated immunodeficiency syndrome	TB	224	BALF, Blood (mNGS)	Fever, cough, expectoration, anorexia, shortness of breath, weight loss, swollen lymph nodes, rash, pleural effusion	Mass shadow, burr sign, cavity, patchy, nodular, and cord-like increased density shadows, multiple enlarged lymph nodes in the left hilum, mediastinum, bilateral heart and diaphragm angles, left pleural effusion	Purulent secretions from the left bronchus, the mucosa of the left upper lobe is obviously thick and rough, the lumen is narrow, and the purulent secretions are attached	Abnormal concentration of imaging agent in the skull, thoracic spine, lumbar spine, and ribs	AMB 0.6 mg/kg/day for 2w, in combination AMB 50 mg/d administered by inhalation , and AMB instillation under bronchoscopy	VCZ for 3 months, ITC for 2 months	Improved
P10	Ant anti-IFN-γ autoantibody associated immunodeficiency syndrome, Sweet’s Syndrome	None	288	Sputum(c), BALF(mNGS), cervical lymph nodes(NGS) *Mycobacterium abscessus*	Rash, joint pain, headache	Cord-like, patchy shadows, nodular shadows	The mucosa of the lower segment of the left main bronchus and the opening of the left upper and lower lobes had uneven hypertrophic nodular protrusions, plasmatic secretions	Skull, right humerus, bilateral femur, bilateral tibia, abnormal concentration of imaging agent	VCZ 200 mg every 12 h for 2 w	VCZ for 1y	Death
P11	An anti-IFN-γ autoantibody-associated immunodeficiency syndrome, Type 2 diabetes mellitus	Lung cancer	47	BALF(c + mNGS), sputum(c)	Cough, hoarseness, fatigue, anorexia, anaemia, weight loss, pleural effusion	Mass shadow, nodule, cord shadow, mediastinal swollen lymph nodes. Right pleural thickening, pericardial effusion	Mucosal congestion and oedema, the left main bronchus and the lower part of the right intermediate trunk were all seen with protruding lumps on the mucosal surface, left main bronchus lumen and right intermediate trunk lumen had stenosis	Abnormal concentration of imaging agent in the right seventh anterior rib and bilateral knee joints	VCZ 200 mg every 12 h for 2 w	VCZ for 15 months	Improved
P12	An anti-IFN-γ autoantibody-associated immunodeficiency syndrome	Lung cancer	139	Bronchial mucosa, BALF(c)	Cough, expectoration, weight loss, swollen lymph nodes, pleural effusion	Mass shadow, patch shadow, nodular increased density shadow, bronchial stenosis, occlusion, multiple enlarged lymph nodes in bilateral hilum and mediastinum, left pleural thickening, left pleural effusion, pericardial effusion	Mucosal congestion, left main bronchus nodules, rough mucosa, tumour blocking the lumen, necrosed tissue attached to the surface of the tumour, stenosis of the left main bronchus lumen, and a lot of yellow purulent secretions	Abnormal concentration of imaging agent in the right seventh anterior rib and bilateral knee joints	VCZ 200 mg every 12 h, and in combination AMB 0.6 mg/kg/day for 1w, AMB 50 mg/d administered by inhalation	VCZ for 1 month, then ICZ for 10 months	Death
P13	An anti-IFN-γ autoantibody-associated immunodeficiency syndrome	None	202	BALF, skin lesion(c), BALF(NGS)*Mycobacterium intracellulare*	Rash, cough, expectoration, weight loss, swollen lymph nodes, pleural effusion	Mass shadow, patchy, cord-like increased density shadow, consolidation shadow, bronchial stenosis, hilar and mediastinal enlarged lymph nodes	The right middle and lower lobe mucosa were hypertrophic, congested and oedematous, and round nodules in the medial segment of the right middle lobe completely blocked the lumen	Skull, sternum, multiple ribs, left ilium, right femur, and bilateral ankle joints had small patches of an abnormal concentration of imaging agent	AMB 0.6 mg/kg/day for 2 months	VCZ for 7 months, then ICZ for 6 months	Improved
P14	Ankylosing spondylitis, Anti-IFN-γ autoantibody associated immunodeficiency syndrome	Lung cancer	16	BALF(c), lung tissue(NGS) Mycobacterium Columbia	Cough, fever, chest tightness, lower extremity oedema, pleural effusion	Mass shadow, patchy, streak shadow, obstructive pneumonia, bronchial occlusion, left parahilar enlarged lymph node, left pleural effusion	The left upper and lower lobe mucosa had hyperaemia and oedema. The left lower lobe mucosa was swollen, hypertrophic and hyperplastic, with a local nodular bulge, the openings of the left lower lobe dorsal and basal segments were narrowed and blocked, purulent discharge	ND	FLC 400 mg/day for 10 days, then changed to VCZ 200 mg every 12 h 4 weeks	None	Death
P15	Ankylosing spondylitis, Anti-IFN-γ autoantibody associated immunodeficiency syndrome	Bacterial pneumonia	134	BALF(NGS), BALF(NGS) Mycobacterium tuberculosis	Cough, expectoration, anorexia, chest pain, weight loss, swollen lymph nodes, pleural effusion	Cord shadow, enlarged lymph node shadow in hilum and mediastinum, pericardial effusion	Multiple small nodules and white secretions can be seen in the openings of both main bronchi, right middle lobe, left proprioceptive upper lobe, left lingual lobe and left lower lobe	The ribs, vertebral bodies, left iliac spine, and left acetabulum showed abnormally concentrated small patches of the imaging agent	VCZ 200 mg every 12 h, and in combination AMB 0.6 mg/kg/day for 1w	None	Death
P16	Anti-IFN-γ autoantibody-associated immunodeficiency syndrome	Lung cancer, lymph node tuberculosis	104	BALF(c + NGS), Blood culture positive for Salmonella typhi	Fever, cough, expectoration, wheezing, swollen lymph nodes, pleural effusion	Patchy shadow, bronchial occlusion, enlarged mediastinal lymph nodes, left prethoracic effusion	In the opening of the left main bronchus, the nodules were fused, and the mucosa around the surface was covered with necrosis, showing scaly hypertrophy, thick and rough mucosa, and a narrow lumen	On the left lateral orbital rim, multiple ribs, sternum, pelvis, and the middle and upper segment of the right femur, there were spot-like abnormal concentration of imaging agent	VCZ 200 mg every 12 h 1 week, and in combination AMB 50 mg/d administered by inhalation, and AMB Instillation under bronchoscopy	VCZ for 3 months, then ICZ for 13 months	Cured
P17	An anti-IFN-γ autoantibody-associated immunodeficiency syndrome	Lung cancer	124	BALF(c), Blood(NGS)	Cough, expectoration, chest pain, shortness of breath, weight loss, pleural effusion	Mass shadow, patch shadow, cord shadow, nodule shadow, bronchial stenosis, occlusion, hilar, mediastinal lymph node enlargement, right pleural effusion	A round nodular mass was seen at the opening of the right middle and lower lobe	No exception found	AMB 0.6 mg/kg/day for 10 ds, change to VCZ 200 mg every 12 h 3 weeks in combination with AMB 50 mg/d administered by inhalation	VCZ for 3 months, then ICZ for 13 months	Improved
P18	Anti-IFN-γ autoantibody-associated immunodeficiency syndrome	Lung cancer	109	BALF(c)	Cough, chest pain, shortness of breath after activity	Patchy, cord-like high-density shadow, pleural traction, depression, burr sign, nodular shadow, endobronchial nodules. hilar, mediastinal lymph node enlargement	New organisms with haemorrhages can be seen in the right middle lobe. The lumen is approximately 50% blocked	The 1st anterior rib on the right, the 11th thoracic vertebra, the 8th posterior rib on the left, the 4th lumbar vertebra, and the left forearm showed irregular patchy imaging agent concentrations	VCZ 200 mg every 12 h 2 weeks	VCZ for 16 months, then ICZ for 7 months	Improved
P19	Anti-IFN-γ autoantibody-associated immunodeficiency syndrome	Lung cancer	55	BALF(c + NGS), Blood culture positive for Salmonella typhi	Cough, expectoration, chest pain, shortness of breath, weight loss, pleural effusion	Patchy and cord-like increased density shadows, obstructive pneumonia, multiple enlarged lymph nodes in both hilum, mediastinum and right supraclavicular fossa, left pleural effusion, pericardial effusion	The mucosa of the right middle lobe is uneven, hypertrophic, and rough. New organisms and purulent secretions were seen in the middle of the left main bronchus	Abnormal concentration of imaging agent in the right sacroiliac joint	AMB 0.6 mg/kg/day for 2 weeks, and in combination with AMB Instillation under bronchoscopy	VCZ for 7 months	Cured

ND: Not done; *PR*: present report; AMB: Amphotericin B; ICZ: Itraconazole; FLZ: Fluconazole; VCZ: Voriconazole; TB: Tuberculosis.

Diagnostic methods to demonstrate *T. marneffei* infection were culture (c), histopathology (h), and metagenomic next-generation sequencing (mNGS).

### Laboratory examination results

Assessments of complete blood count results revealed increased levels of white blood cells and neutrophils in all patients but decreased haemoglobin concentrations and platelet counts in 15 patients and 10 patients, respectively. Serum biochemical analysis revealed that serum albumin concentrations were below the normal range in all patients. The C-reactive protein concentration, erythrocyte sedimentation rate, and procalcitonin (PCT) level were increased in all patients. The serum GM test was performed in 19 patients, but positivity was observed in only 2 patients. The G test was performed in 18 patients, and only one patient had elevated levels (positive: > 100 pg/mL). Moreover, 7 patients exhibited positivity in the bronchoalveolar lavage fluid (BALF) GM test, and 4 patients exhibited positivity in the G-test. Ten patients showed increased globulin levels, and serum IgG levels were increased in 12 patients. Serum IgM levels were increased in 7 patients and decreased in 3 patients. The CD4 and CD8 lymphocyte counts were determined in 16 patients by flow cytometry and were decreased in two patients (Table 2).

**Table 2.** Laboratory work-up results for tracheobronchial *T. marneffei* infection.

Laboratory examination	P1	P2	P3	P4	P5	P6	P7	P8	P9	P10	P11	P12	P13	P14	P15	P16	P17	P18	P19
WBC(×10^9^)	14.3	17	9.42	12.5	19.83	17.5	12.88	7.16	34.09	29.79	24.45	21.57	9.91	15.31	22.66	18.92	19.67	21.45	7.86
ANC(×10^9^)	11.4	12.7	6.09	8.4	18.18	15.1	8.8	5.56	26.86	26.35	21.99	18.09	4.8	12.43	0.894	14.24	17.2	17.29	5.21
ALC(×10^9^)	1.3	2.7	2.03	3.4	0.78	0.9	2.1	0.8	2.69	1.97	1.83	2.19	4.03	1.59	1.18	2.52	1.1	2.67	1.88
HGB(g/L)	72	91	119	75	94.1	93	106.5	126.5	88.2	97	65.1	148	63.5	107.9	89.4	85.6	111.6	141	96
PLT(×10^9^)	648	528	409	160	514.8	367	227.6	326.3	578.7	538.4	459.2	360	502.8	247.3	575.6	464.3	236	283	377.6
Albumin (g/L)	25.9	23.5	32.8	21.1	22	28.4	29	31.6	22.6	24.1	29.2	30.7	30.1	26.6	22.9	22.5	29.7	37.9	35.2
Globulin (g/L)	59.1	49.2	55	46.6	52.7	28.3	28.2	27.9	44.9	44	67.2	39.6	57.1	38.5	39.8	43.4	32.9	37.1	30.5
ALT(u/L)	9	23	8	18	8	24	20	12	19	36	15	20	7	65	11	8	32	37	11
AST(u/L)	14	11	9	22	20	50	22	19	11	23	16	16	11	47	34	14	22	21	11
CRP(mg/L)	48.99	88.63	7.68	92.33	34.04	171.18	86.58	>192	166.04	114.84	91.2	112	159.65	99.05	137.23	143	190.68	19.63	70.86
ESR(mm/h)	112	108	<140	108	100	85	53	66	102	96	>120	65	102	85	50	98	76	58	108
PCT(ng/L)	ND	0.149	ND	0.314	0.505	NM	1.39	0.293	0.631	0.209	ND	0.231	0.151	0.24	0.334	0.32	2.73	0.122	0.047
G-test(pg/mL)(serum)	ND	<10	80.5	95.5	<10	<10	<10	<10	<10	<10	421.39	<10	<10	<10	<10	<10	<10	51.49	<10
GM-test(serum)	0.523	0.164	0.543	0.224	0.222	0.326	0.238	0.592	0.195	0.326	0.344	0.279	0.353	0.153	0.339	0.389	0.3	0.388	0.392
G-test(BALF)	<10	<10	166.3	<10	95	<10	ND	<10	<10	<10	<10	200.09	<10	280.8	56.77	52.6	34.26	33	223.7
GM-test(BALF)	0.230	0.338	1.007	0.663	0.296	0.326	ND	0.678	0.243	2.824	0.467	0.886	0.235	0.363	0.199	0.227	1.663	0.866	0.237
IgG(g/L)	36.09	30.76	35.15	38.4	24.34	10.09	22.36	8.44	ND	19.73	49.62	19.14	ND	22.96	20.37	26.57	14.15	15.78	12.99
IgA(g/L)	ND	1.631	2.119	NM	3.34	1.506	4.73	0.95	ND	4.57	3.54	1.81	ND	3.58	2.67	3.62	1.89	2.57	1.67
IgM(g/L)	ND	2.595	0.539	NM	0.73	1.562	1.16	0.32	ND	0.99	2.46	1.64	ND	1.52	1	1.47	1.08	2.27	1.14
CD4^+^T cell count(cells/μL)	325	1164	744	969	ND	425	1202	305	ND	1478	519	921	755	ND	538	1226	474	953	674
CD8^+^T cell count (cells/μL)	393	1164	576	1470	ND	282	518	327	ND	600	471	748	1059	ND	245	1116	451	848	703
CD4%	ND	937	39.9	25.9	23	34.5	ND	28.51	29.19	52.92	35.01	38.63	28.93	25.23	41.5	41	31.54	36.87	32.6
CD8%	ND	ND	24.7	39.3	34.5	22.9	ND	30.63	34.08	21.48	31.81	31.38	40.59	17.45	18.9	37.3	30	32.82	34

ND Not done; NM not mentioned; BALF bronchoalveolar lavage fluid.

Normal ranges: C-reactive protein: < 10 mg/L; erythrocyte sedimentation rate: ≤ 15 mm/h; immunoglobulin (Ig) IgG: 8–18 g/L; IgA: 0.9–4 g/L; IgM: 0.84–1.32 g/L; CD4 + T cell count: 410–1590 cells/μL; CD8^+^ T cell count: 190–1140 cells/μL; CRP: C-reactive protein; ESR: erythrocyte sedimentation rate; IgG: serum immunoglobulin G; IgA: serum immunoglobulin A; IgM: serum immunoglobulin M.

### Imaging examination results

Chest CT indicated that all patients had different pulmonary lesions. The most common features of CT images were patchy exudates (*n* = 16, 84.2%), fibrous cords (*n* = 12, 63.2%), pleural thickening (*n* = 7, 36.8%), consolidation and nodular shadows (*n* = 6, 31.6%), intrapulmonary mass shadow (*n* = 5, 26.3%), obstructive pneumonia (*n* = 5, 26.3%), and a cavity (*n* = 3, 15.8%). Moreover, there were 5 cases of tracheobronchial stenosis, 3 cases of bronchial occlusion, and 1 case of bronchial nodules. There were 14 cases of pleural effusion, 14 cases of hilar and mediastinal lymphadenopathy, and 3 cases of pericardial effusions (Figure 1).

Emission CT (ECT) was performed in 11 patients and revealed significantly increased uptake in multiple bones. Multiple instances of abnormal imaging agent concentrations or bone destruction were observed in the skull, clavicle, sternum, femur, humerus, thoracic spine, lumbar spine, pelvis, ribs, ilium, shoulder joint, sacroiliac joint, knee joint, and ankle joint (Figure 1).

### Endoscopy results

All patients underwent fibreoptic bronchoscopy. There were 13 neoplasms (5 masses, 7 polyps/nodules, and 1 bulge), 6 cases of mucosal oedema/hypertrophy, 4 cases of mucosal unevenness, 8 cases of purulent secretions, and 8 cases of bronchial stenosis/occlusion. The lesions involved were as follows: 1 in the trachea; 19 in the left bronchus, including 8 in the left main bronchus, 5 in the left upper lobe bronchus, and 6 in the left lower lobe bronchus; and 18 in the right bronchus, including 2 in the right main bronchus, 2 in the right upper lobe bronchus, 10 in the right middle lobe bronchus, and 4 in the right lower lobe bronchus. In addition, 12 patients exhibited involvement of 2 or more regions (Figure 1).

### Fungal culture and histopathology results

Ten cases of tracheobronchial *T. marneffei* infection were identified *via* positive specimen culture. One case was identified by histopathological examination and culture, 5 cases were confirmed by culture combined with mNGS, and 2 cases were confirmed by mNGS. In addition, seven patients were diagnosed with *T. marneffei* infection by histopathology of the bronchial mucosa. Before and after *T. marneffei* infections, 7 patients (36.8%) were also infected with other opportunistic pathogens, including NTM (*n* = 3; 15.8%), *Salmonella* spp. (*n* = 2; 10.5%), *Mycobacterium tuberculosis* (*n* = 1; 5.3%), and *Staphylococcus haemolytic* (*n* = 1; 5.3%).

### Treatments and outcomes

For the initial induction therapy, 7 patients were initially treated with voriconazole, 6 patients were initially treated with amphotericin B, and 2 patients were initially treated with voriconazole combined with amphotericin B. All patients were sequentially treated with voriconazole 0.2 g q12 or itraconazole 0.2 g BID orally. One patient was initially treated with fluconazole, followed by itraconazole, and was finally cured. Three patients were initially treated with fluconazole, but the curative effect was not good. They were switched to voriconazole 0.2 g q12 and amphotericin B 0.6 mg/kg/d, and their symptoms improved. The treatment in 5 patients also involved nebulization (25 mg BID) and/or instillation of amphotericin B under fibreoptic bronchoscopy. The total treatment time was 12 months (8, 15 months), and 2 patients continued to take antifungal drugs for more than 3 years. Two patients received active antifungal treatment immediately after diagnosis but eventually died of multiple organ dysfunction syndrome (MODS), while 3 patients had good initial treatment effects, and the total antifungal course of treatment was more than 10 months. Three patients were treated with anti-NTM medication. According to the drug susceptibility results, 3–4 drugs, including moxifloxacin, clarithromycin, ethambutol, imipenem, and linezolid, were combined with anti-infective treatment, and the total course of treatment was not less than 12 months. One patient received paclitaxel combined with carboplatin chemotherapy for underlying lung cancer. To date, 6 patients were cured, 8 patients improved, and 5 patients (26.3%) died.

## Discussion

Talaromycosis marneffei is a severe invasive disseminated fungal disease caused by *T. marneffei*, which is mainly prevalent in Southeast Asian countries and Southern China [4]. Inhaling *T. marneffei* spores from the environment and their passage through the respiratory tract may be the key mode of transmission. It has also been clinically observed that HIV-negative *Talaromycosis marneffei* patients with immunodeficiency are most likely to exhibit respiratory system involvement, which occurs earliest in these patients, and the highest rate of *T. marneffei* isolation is from respiratory and lung tissue specimens [1]. However, unlike pulmonary involvement, which is easy to diagnose, tracheobronchial involvement is difficult to detect clinically, clinical reports are extremely rare, and bronchoscopy can be performed only when dyspnoea or fatal asphyxia occurs. Therefore, the systematic summary and analysis of its clinical characteristics reported here have clinical importance.

The 19 patients with tracheobronchial *Talaromycosis marneffei* included in this study were all HIV-negative patients, and 14 were farmers who had been engaged in agricultural activities for a long time. Common clinical features included fever, lymphadenopathy, cough, and dyspnoea. Tracheobronchial *Talaromycosis marneffei* is often misdiagnosed as pulmonary tuberculosis or lung cancer. The misdiagnosis rate is as high as 68.4% because misdiagnosis leads to inappropriate treatment and to a prolonged course of disease. In this study, the time from onset to diagnosis was as long as 133 d. According to the literature, the detection rate of *T. marneffei* infection of HIV-negative patients, the time of diagnosis and timely treatment are the main features underlying the clinical prognosis. The detection rate of pathogens associated with *Talaromycosis marneffei* in AIDS patients is high in clinical specimens, and these infections are easy to diagnose. After being diagnosed with AIDS, these patients need to be transferred to a special AIDS hospital for timely treatment according to China’s epidemic prevention requirements. Therefore, this study did not include HIV-positive hosts. Data show that AIGA-related immunodeficiency, autoimmune diseases, application of glucocorticoids and/or immunosuppressive agents, and malignant tumours are the most common immune disorders in HIV-negative *T. marneffei*-infected patients [5]. The most common is an anti-cytokine disease, such as AIGA-related immunodeficiency, accounting for 25.1% [5]. Studies have shown that the AIGA titre is an independent risk factor for *T. marneffei* infection and recurrence as well as multiple infections [5]. Immunodeficiency caused by AIGA is an emerging adult-onset immunodeficiency syndrome first described in 2004 [6]. INF-γ plays a very important role in the body’s defence against intracellular pathogen infection. Increased antibody titres can inhibit the signal transduction of INF-γ and the production of TNF-α and IL-12, resulting in severely impaired Th1 responses. There is also increased susceptibility of the body to pathogens, especially intracellular infectious pathogens, such as *T. marneffei*, NTM, nontyphoid *Salmonella*, *Burkholderia cepacia*, varicella-zoster virus, cytomegalovirus [7]. Studies have demonstrated that autoantibodies against IFN-γ are the main aetiology of *T. marneffei* infections in HIV-negative individuals, in which the risk HLA–class II alleles HLA-DRB1*16:02 and -DQB1*05:02 are highly prevalent [8] There were 11 patients (57.9%) with increased AIGA levels in this group. Therefore, autoantibodies against IFN-γ may predispose these patients to *T. marneffei* infection [9].

The 19 *Talaromycosis marneffei* patients in this study showed prominent clinical symptoms of respiratory system involvement, such as cough, expectoration, chest tightness, shortness of breath, and lung shadows, accompanied by systemic inflammatory responses that were manifested as fever, anaemia, weight loss, hypoalbuminemia, significantly increased levels of WBCs and neutrophils, elevated platelet counts, and varying degrees of increased anaemia and levels of the inflammatory indicators ESR, CRP, and PCT. The rates of positivity for G and GM antigens in bronchoalveolar lavage fluid were higher than those in peripheral blood. The pathological findings in sites of airway involvement included purulent changes dominated by neutrophil infiltration, which was accompanied by the formation of chronic granulomas. In this study, all the affected tissue specimens, including blood, lymph node, and skin tissue samples, obtained from the patients were sent to be examined for aetiology. Respiratory tract specimens, including mucosal tissue and bronchoalveolar lavage fluid, had the highest rate of positivity for *T. marneffei* isolation, and the diagnosis methods included routine culture and pathological tissue assessments. MNGS examination was performed on 7 specimens. The mNGS detection results in 6 patients were consistent with the results of conventional detection methods, but the diagnosis time was advanced by more than 1 week. In addition, 2 patients with *T. marneffei* infection had confirmed diagnoses only with mNGS but exhibited negativity in conventional culture, suggesting that mNGS is a sensitive detection method. In addition, 3 patients whose infections were combined NTM infections, especially slow-growing NTM infections, were identified using mNGS, which indicates that the use of mNGS can reduce the chance of early missed diagnosis. The mNGS assessment has obvious advantages for immunosuppressed hosts, those clinically suspected to exhibit multiple infections, and those with intracellular bacterial infections with a low rate of positivity in routine culture including *T. marneffei* [10]. For HIV-negative Talaromycosis hosts, 94.8% of patients have immunodeficiency caused by high titres of AIGA [8] ,so the possibility of infection with other opportunistic pathogens is high. Bronchoscopy is recommended for all patients with Talaromycosis with respiratory symptoms, with or without abnormal lung manifestations. In addition to obtaining an early understanding of whether the respiratory tract is involved, the obtained respiratory tract specimens can be evaluated as soon as possible, and corresponding treatment plans can be formulated. All patients in this study showed diverse results in assessments of abnormal chest imaging findings, including multiple patchy densities or consolidation shadows; intrapulmonary mass-like lesions or cavities; mediastinal and/or hilar lymph node enlargement; ground glass opacity; diffuse or miliary nodules; and pleural or pericardial effusion. During bronchoscopy, nodules, masses, and new organisms were observed in the involved airways, which could block the bronchial lumen or cause lumen stenosis, accompanied by mucosal congestion, hypertrophy, and purulent secretions in the airways. Rare tumours or mucosal erosions, ulcers, and other features were also observed. *T. marneffei* infections can involve the trachea, main bronchi, lobar bronchi, and segmental bronchi, with the bronchi being more commonly involved than the trachea. These microscopic manifestations are similar to those of bronchial lung cancer, bronchial aspergillosis, and tracheal tuberculosis, which are difficult to differentiate under the microscope. Thus, *T. marneffei* infections must be diagnosed by histopathology and pathogenic bacterial examination. If malignant tumour cells are found, *T. marneffei* infection may be diagnosed as a tumour, but if a chronic granuloma is identified, the disease may be tuberculosis or may be due to an Aspergillus infection, an NTM infection, a *T. marneffei* infection, or other infections. Special staining, such as acid-fast staining, PAS staining, and D-PAS staining, as well as molecular biotechnology approaches, such as culture and mNGS, are used to detect and identify pathogens. We found *T. marneffei* in lung cancer tissue in 1 patient in this group, and *T. marneffei* was found in the bronchoalveolar lavage fluid, blood, and bronchial mucosal tissue of 7 patients. It is also an opportunistic infection often associated with other intracellular pathogens.

For disseminated *Talaromycosis marneffei*, antifungal therapy should be based on systemic therapy. *In vitro* experiments show that yeast-phase *T. marneffei* has high sensitivity to amphotericin B, posaconazole, voriconazole, and itraconazole; it is also sensitive to fluconazole to a certain extent. However, during clinical treatment, drug resistance can be induced [11]. At present, there is no standard treatment for Talaromycosis in HIV-negative hosts. Clinical treatment guidelines often refer to the treatment of HIV-positive Talaromycosis patients and include the intravenous infusion of amphotericin B (0.6 mg/kg d) followed by oral itraconazole (400 mg/d) maintenance therapy [12]. Voriconazole is a second-generation triazole antifungal drug with a definite effect on *T. marneffei* infections. It can be the first-choice treatment or used in patients who cannot tolerate amphotericin B [13]. The patients in this group were treated with amphotericin B, itraconazole, and voriconazole for antifungal therapy. If the initial treatment with fluconazole was not effective, voriconazole or amphotericin B was administered, which exhibited satisfactory clinical efficacy. The total treatment time in most of the patients was more than 1 year; 2 patients were treated with antifungal therapy for more than 3 years, and they are still being monitored in follow up. We reported a case of *T. marneffei* infection involving the main tracheal structure that was treated with only intravenous antifungal drugs. Although the effect was obvious in some regions, such as the lungs, airway cartilage and areas of tube wall destruction, pathologies including airway collapse, asphyxia, and other serious complications were observed [2]. It has been suggested that the use of systemic antifungal therapy alone is not sufficient in *Talaromycosis marneffei* patients with tracheobronchial involvement. Therefore, we recommend the use of nebulized inhalation or intratracheal instillation of amphotericin B in the treatment of invasive pulmonary aspergillosis [14]. In our study, 5 patients were treated with the combined use of intravenous administration of effective antifungal drugs and local nebulized inhalation of amphotericin B (25 mg BID for 1 month); this treatment strategy was associated with the rapid absorption of the drug into the lung, trachea and bronchial lesions and no adverse reactions. The indications for nebulization therapy include *Talaromycosis marneffei* patients with tracheal, bronchial or lung tissue involvement confirmed by imaging or bronchoscopy. Combined local therapy is particularly important, especially when the disease is complicated with obstructive atelectasis, tracheal stenosis, and other complications. When a combination of infections is identified that includes opportunistic nonfungal infections, it is necessary to choose the appropriate treatment plan for the different pathogens involved. In addition, treatment of coexisting basic diseases, such as lung cancer, should also be included.

## Conclusions

In short, *T. marneffei* involvement of the airway should be considered an important clinical feature of *Talaromycosis marneffei*. However, the clinical understanding is not sufficient, so missed diagnoses are rarely reported. Early bronchoscopy has important clinical value for the early diagnosis and treatment of *T. marneffei*. Nebulized inhaled amphotericin B and/or bronchoscopy drops may have better efficacy.

## Supplementary Material

Supplemental Material

## Data Availability

The authors confirm that the data supporting the findings of this study are available within the article and its supplementary materials.
