# Supplementary material for: Clinical characteristics of tracheobronchial Talaromyces marneffei infection in non-HIV-infected patients in South China
Source: Ann Med. 2023 Nov 15;55(2):2276310. doi: 10.1080/07853890.2023.2276310 (PMC10653738; doi:10.1080/07853890.2023.2276310)
Supplement: Supplemental Material [file IANN_A_2276310_SM9752.docx]

Table Characteristics of the 19 patients with tracheobronchial *T. marneffei* infection.

| Sex |  |  |
| --- | --- | --- |
| Male (%) |  | 11 (57.89) |
| Female (%) |  | 8 (42.11) |
| Age (years) (median, IQR) |  | 52 (45, 62) |
| Main presenting symptoms |  |  |
| Cough (%) |  | 18（94.7） |
| Expectoration (%) |  | 17（89.5） |
| Fever (%) |  | 14（73.7） |
| Weight loss (%) |  | 10（52.6） |
| Anaemia (%) |  | 16 (84.21) |
| Imaging examination |  |  |
| Patchy exudates (%) |  | 16 (84.2) |
| Fibrous cords (%) |  | 12( 63.2) |
| Pleural thickening (%) |  | 7 (36.8) |
| Consolidation and nodular shadows (%) |  | 6 (31.6) |
| Intrapulmonary mass shadow (%) |  | 5 (26.3) |
| Obstructive pneumonia (%) |  | 5 (26.3) |
| Cavity (%) |  | 3 (15.8) |
| Pleural effusion (%) |  | 14 (73.68) |
| Hilar and mediastinal lymphadenopathy (%) |  | 14 (73.68) |
| Pericardial effusions (%) |  | 3(33.33) |
| Endoscopy |  |  |
| Neoplasms (%) |  | 13 (68.42) |
| Mucosal oedema/hypertrophy (%) |  | 6 (31.58) |
| Mucosal unevenness (%) |  | 4 (21.05) |
| Purulent secretions (%) |  | 8 (42.11) |
| Bronchial stenosis/occlusion (%) |  | 8 (42.11) |
| Diagnostic methods |  |  |
| Culture |  | 10 |
| Histopathology |  | 1 |
| Histopathological examination+ culture |  | 1 |
| Culture combined+ mNGS |  | 5 |
| mNGS |  | 2 |
| First-line anti-fungal therapy |  |  |
| Voriconazole (%) |  | 7(36.8) |
| Amphotericin B (%) |  | 6 (31.6) |
| Voriconazole combined with amphotericin B (%) |  | 2(10.53) |
| Fluconazole (%) |  | 1(5.26) |
| Fluconazole, switched to voriconazole or amphotericin B (%) |  | 3(33.33) |
| Outcome |  |  |
| Cured (%) |  | 6 (31.58) |
| Improved (%) |  | 8 (42.11) |
| Died (%) |  | 5 (26.3) |
